# Molecular signaling networks that connect tissue dysfunction to systemic insulin resistance

**DOI:** 10.3389/fendo.2026.1952238

**Published:** 2026-10-06

**Authors:** Rashid Mir, Naseh A. Algehainy, Mohammed M. Jalal, Malik A. Altayar, Reema M. Almotairi, Faris J. Tayeb, Ruqaiah I. Bedaiwi, Faisal H. Altemani, Kholoud S. Almasoudi, Ibrahim Altedlawi Albalawi, Imadeldin Elfaki

**Affiliations:** 1 Prince Fahad Bin Sultan Chair for Biomedical Research, University of Tabuk, Tabuk, Saudi Arabia; 2 Department of Medical Laboratory Technology, Faculty of Applied Medical Sciences, University of Tabuk, Tabuk, Saudi Arabia; 3 Department of Surgical Oncology, Faculty of Medicine, University of Tabuk, Tabuk, Saudi Arabia; 4 Department of Biochemistry, Faculty of Science, University of Tabuk, Tabuk, Saudi Arabia

**Keywords:** diabetes, insulin receptor (INSR), metabolic disorders, obesity, protein kinase C, β-cell dysfunction

## Abstract

Insulin resistance is conventionally described as a single condition, but it is better understood as the point at which several tissue-specific signaling failures, each following its own molecular logic and its own timeline, converge into one measurable phenotype. This review treats insulin resistance as a network rather than a collection of independent organ pathologies. Because the components of this network rest on very different kinds of evidence, each node and edge is graded explicitly by study design and causal status, and the time-staged sequence is presented as a falsifiable proposal rather than an established progression. Put simply, insulin resistance is treated here not as one defect but as the shared endpoint of several tissue-specific failures that develop at different times and communicate with one another. Liver, skeletal muscle, adipose tissue, pancreatic islets, the gut, and the brain are each examined as nodes with characteristic signaling lesions built from a shared architecture of diacylglycerol- and ceramide-activated kinases, inhibitory serine phosphorylation of insulin receptor substrate proteins, and endoplasmic reticulum stress, while inter-organ messengers, incretins, adipokines, bile acids, and autonomic circuits, are treated as the edges that couple these nodes together. The pancreas is included not as a site of resistance but as the network’s compensatory arm, and the brain is treated as an integrated hypothalamic-vagal node rather than a separate neurocognitive topic, closing a loop in which adipose-derived signals impair central insulin and leptin action, weakened central signaling relaxes hepatic glucose control, and reduced satiety sustains the intake that drove adipose dysfunction in the first place. A dedicated section addresses the post-translational and organelle-level machinery, shared serine kinases, ubiquitin-mediated IRS degradation, O-GlcNAcylation, and mitochondria-endoplasmic reticulum contact sites, that allows the same small set of molecular tools to produce different tissue-specific outputs. These mechanisms are then placed on an explicit time axis moving from silent compensation, in which adipose and hypothalamic changes precede any measurable defect elsewhere, through early decompensation in liver and muscle, to established decompensation marked by β-cell failure and self-sustaining feed-forward loops, a staging distinction that is largely absent from mechanistic studies conducted at a single, typically late, disease stage. This framing helps explain a pattern in current therapeutics: interventions that act on several network nodes at once, most notably incretin-based therapies, have outperformed mechanistically well-motivated single-node strategies, including direct inhibition of shared convergence kinases. The review closes by identifying where the network remains least understood, principally in longitudinal, tissue-resolved human data capable of testing the proposed staging sequence directly.

## 1 Introduction

Insulin resistance is usually described as a single condition, but it is better understood as an outcome — the point at which several tissue-specific signaling failures converge to produce one measurable phenotype: a blunted response to insulin at the whole-body level. Throughout this review, “insulin resistance” refers to this whole-body, physiologically defined reduction in insulin action unless a specific molecular signaling defect or a clinical diagnosis of type 2 diabetes (T2D) is explicitly indicated; these three levels—molecular, physiological, and clinical—are related but not equivalent, and we flag which is meant where the distinction matters. Liver, skeletal muscle, adipose tissue, pancreatic islets, the gut, and the brain each develop their own characteristic molecular lesions, on their own timelines, and only later do these local failures add up to the systemic picture that clinicians call insulin resistance. This distinction matters, because much of the existing literature has organized the topic either around the cell-intrinsic signaling cascade downstream of the insulin receptor, or around the circulating messengers (lipids, cytokines, hepatokines, myokines, adipokines, exosomal microRNAs) that carry information between organs (1, 2). Considerably less has been written about how the intracellular signaling architecture inside each tissue is itself reorganized — rewired, not just dampened — as insulin resistance develops, and how that tissue-specific rewiring proceeds through a recognizable sequence, from an initial compensated state through progressive decompensation.

This review takes the network as its organizing unit rather than the organ or the pathway. Each tissue is treated as a node with its own characteristic signaling lesion; each inter-organ messenger is treated as an edge connecting nodes; and the whole system is followed forward in time, from the earliest compensatory adjustments to the point at which compensation fails. This framing is not a stylistic preference. Two decades of physiological and multi-omics work have made clear that identical downstream defects — reduced GLUT4 translocation in muscle, unrestrained hepatic gluconeogenesis, unrestrained adipose lipolysis — can be reached by different upstream routes depending on the tissue, the stimulus, and the stage of disease, and that the tissues do not fail in synchrony. Adipose tissue insulin resistance, for example, tends to precede hepatic and muscle insulin resistance during sustained caloric excess (3, 4), and pancreatic β-cells mount a compensatory hyperinsulinemic response that can mask peripheral resistance for years before it exhausts itself (5). A review organized purely by pathway (PI3K–Akt, mTORC1, inflammatory kinases) risks treating these temporal and tissue-specific features as incidental detail; a review organized purely by organ risks losing the shared molecular machinery — post-translational modifications, organelle contact sites — that couples the nodes together. Here, both are kept in view together. Six tissues make up this network. The first three, liver, skeletal muscle, and adipose tissue, are the classical targets of insulin action and account for the majority of insulin-stimulated glucose disposal and suppression of glucose output. The pancreas is included not as a site of insulin resistance in the conventional sense, but as the tissue whose β-cell compensation determines whether peripheral resistance remains silent or becomes hyperglycemia — the compensatory arm of the network that is often set aside in reviews focused solely on insulin target tissues. The gut is included because incretin signaling and microbiota-derived metabolites now sit upstream of both β-cell function and hepatic and adipose signaling, and because this axis has moved from a research curiosity to the mechanistic basis of the most effective current pharmacotherapies. The brain is included as an integrated node in the network rather than as a separate neurocognitive topic, because hypothalamic and hindbrain insulin signaling feed back onto hepatic glucose production and adipose lipolysis through defined vagal and sympathetic circuits (6, 7), making central insulin action a determinant of peripheral insulin sensitivity rather than merely a consequence of it.

The review is organized as follows. Section 2 summarizes the core insulin receptor signaling node — insulin receptor substrate (IRS) proteins, phosphatidylinositol 3-kinase (PI3K), and Akt — that is shared, in modified form, across all six tissues, since the tissue-specific sections that follow can be read as variations on this common template. Sections 3 through 8 examine liver, skeletal muscle, adipose tissue, pancreas, gut, and brain in turn, with attention to the specific signaling lesions documented in each. Section 9 addresses the molecular machinery — post-translational modifications and mitochondria–endoplasmic reticulum contact sites — that operates across tissue boundaries and helps explain why interventions that target one node often have measurable effects in another. Section 10 places these tissue-specific lesions on a common time axis, from early compensation to late decompensation, which is the organizing question that current gaps in the field, including those identified by this Research Topic, converge on. Section 11 considers therapeutic strategies organized by the network node they act on, rather than by drug class alone.

## 2 The core insulin signaling node

Every tissue-specific signaling story in this review begins at the same starting point. Insulin binds the extracellular α-subunits of the insulin receptor (INSR), a transmembrane tyrosine kinase, triggering autophosphorylation of the intracellular β-subunits and creating docking sites for insulin receptor substrate (IRS) proteins, principally IRS-1 and IRS-2 (8, 9). Tyrosine-phosphorylated IRS proteins recruit and activate class IA phosphatidylinositol 3-kinase (PI3K), which converts membrane phosphatidylinositol-4,5-bisphosphate to phosphatidylinositol-3,4,5-trisphosphate and thereby recruits PDK1 and Akt (also called protein kinase B) to the plasma membrane, where Akt is activated by phosphorylation at Thr308 and Ser473 (9). This IRS–PI3K–Akt axis is the shared trunk from which tissue-specific branches diverge; more than one hundred Akt substrates have been described, and which of these substrates matters most in a given tissue depends on that tissue’s physiological job (9).

In skeletal muscle and adipose tissue, Akt phosphorylates the Rab-GTPase-activating protein TBC1D4 (AS160), releasing its inhibition of Rab proteins that control the translocation of GLUT4-containing vesicles to the plasma membrane, and thereby driving insulin-stimulated glucose uptake (10, 11).

In liver, a principal Akt substrate is the transcription factor forkhead box O1 (FOXO1); its phosphorylation excludes FOXO1 from the nucleus and suppresses transcription of the gluconeogenic enzymes phosphoenolpyruvate carboxykinase (PEPCK) and glucose-6-phosphatase, while Akt-mediated inhibition of glycogen synthase kinase 3 (GSK3) relieves inhibition of glycogen synthase and promotes glycogen storage (9). Across liver and adipose tissue, Akt activity, acting through mTORC1 and its downstream effector SREBP-1c, also stimulates *de novo* lipogenesis, so that the same signaling node that suppresses glucose output and promotes glucose storage simultaneously commits carbon toward lipid synthesis (9). This dual output — glucose handling on one branch, lipid handling on the other — is central to understanding insulin resistance, because in most tissues the two branches do not fail together. Hepatic insulin resistance, for instance, is frequently selective: impaired suppression of gluconeogenesis coexists with preserved or even exaggerated SREBP-1c-driven lipogenesis, a pattern described as selective hepatic insulin resistance (12, 13).

Two features of this core node deserve emphasis before moving to individual tissues. First, the node is not a simple on–off switch; its output is graded and shaped by negative feedback loops, including S6 kinase 1 (S6K1)-mediated serine phosphorylation of IRS-1, which under nutrient excess desensitizes the receptor-proximal signal even while distal mTORC1 signaling remains active. Mice lacking S6K1 stay insulin sensitive on a high-fat diet despite obesity-range lipid exposure, with markedly reduced IRS-1 serine phosphorylation, giving this feedback route genetic support (14, 15). This feedback architecture means that chronic nutrient oversupply can produce insulin resistance at the level of glucose uptake while lipogenic and growth signaling remain intact or amplified — again reinforcing that “insulin resistance” is rarely a uniform loss of signal but a redistribution of it. Second, the specific defect that produces reduced glucose uptake or unrestrained glucose output is rarely a mutation or loss of the core proteins themselves; large-scale phosphoproteomic profiling of insulin-resistant tissue has shown that the signaling network is rewired at multiple nodes simultaneously rather than broken at one single point, with altered phosphorylation detected on proteins well outside the canonical IRS–PI3K–Akt trunk (16). This observation is one of the more direct pieces of evidence, from unbiased proteomic data rather than candidate-pathway hypothesis testing, that insulin resistance is a network-level phenomenon rather than a single lesion — the premise on which the rest of this review is built. **Figure 1** summarizes this shared trunk and its four tissue-specific branches.

**Figure 1:**
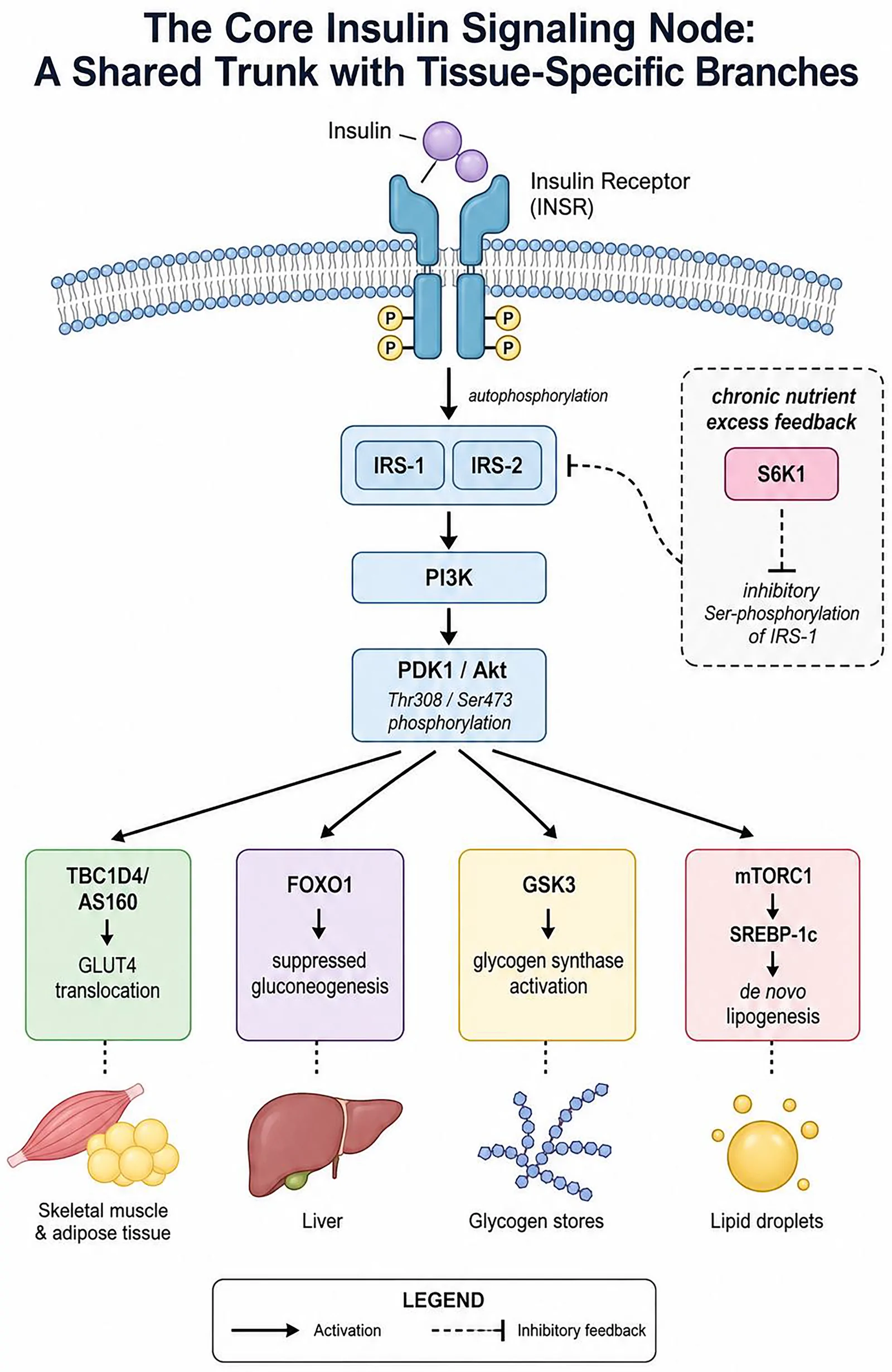
The core insulin signaling node shared across tissues. Insulin receptor autophosphorylation recruits IRS-1/IRS-2, activating PI3K and Akt (phosphorylated at Thr308/Ser473). Akt’s more than 100 substrates diverge into tissue-specific branches: GLUT4 translocation (muscle, adipose), suppression of gluconeogenesis via FOXO1 (liver), glycogen synthesis via GSK3, and lipogenesis via mTORC1/SREBP-1c. Chronic nutrient excess engages S6K1-mediated inhibitory feedback onto IRS-1, providing an early point of dysregulation common to insulin resistance across tissues. All arrows in this figure represent experimentally established biochemical steps.

## 3 Liver: selective resistance and the diacylglycerol–PKCϵ axis

The liver is unusual among the tissues considered here because its insulin resistance is characteristically selective rather than global. Insulin’s suppression of hepatic glucose production is impaired, while its stimulation of *de novo* lipogenesis through SREBP-1c is preserved or enhanced, so that a resistant liver can simultaneously overproduce glucose and overproduce triglyceride (12, 13). Reconciling this pattern with a single shared IRS–PI3K–Akt trunk (Section 2) has been one of the more productive puzzles in the field, and it has been substantially resolved by work identifying a receptor-proximal, rather than a post-Akt, lesion. The best-characterized mechanism centers on hepatic diacylglycerol (DAG). Ectopic accumulation of specific DAG species, generated as a byproduct of incomplete fatty acid oxidation and lipid overflow into the liver, activates the novel protein kinase C isoform PKCϵ, which binds and phosphorylates the insulin receptor at a regulatory threonine residue (Thr1160 in the kinase activation loop), directly reducing insulin receptor tyrosine kinase activity (17, 18). Because this lesion acts at the level of the receptor itself, it plausibly explains why gluconeogenic suppression — which depends on this proximal signal reaching FOXO1 — fails, while SREBP-1c-driven lipogenesis, which can be sustained through Akt-independent and receptor-bypass mechanisms including direct substrate delivery, persists (9, 13).

Human data support this model: dietary interventions that acutely raise hepatic DAG content produce measurable hepatic insulin resistance within hours, well before changes in body weight or adiposity, and the magnitude of PKCϵ activation correlates with the degree of impaired insulin suppression of glucose production in human liver biopsy studies (18, 19). Genetic knockdown of PKCϵ in rodent models normalizes hepatic insulin sensitivity despite unchanged hepatic lipid content, indicating that the DAG–PKCϵ axis rather than steatosis per se is the proximate signaling lesion (17). **Figure 2** lays out these convergent and parallel hepatic routes together.

**Figure 2:**
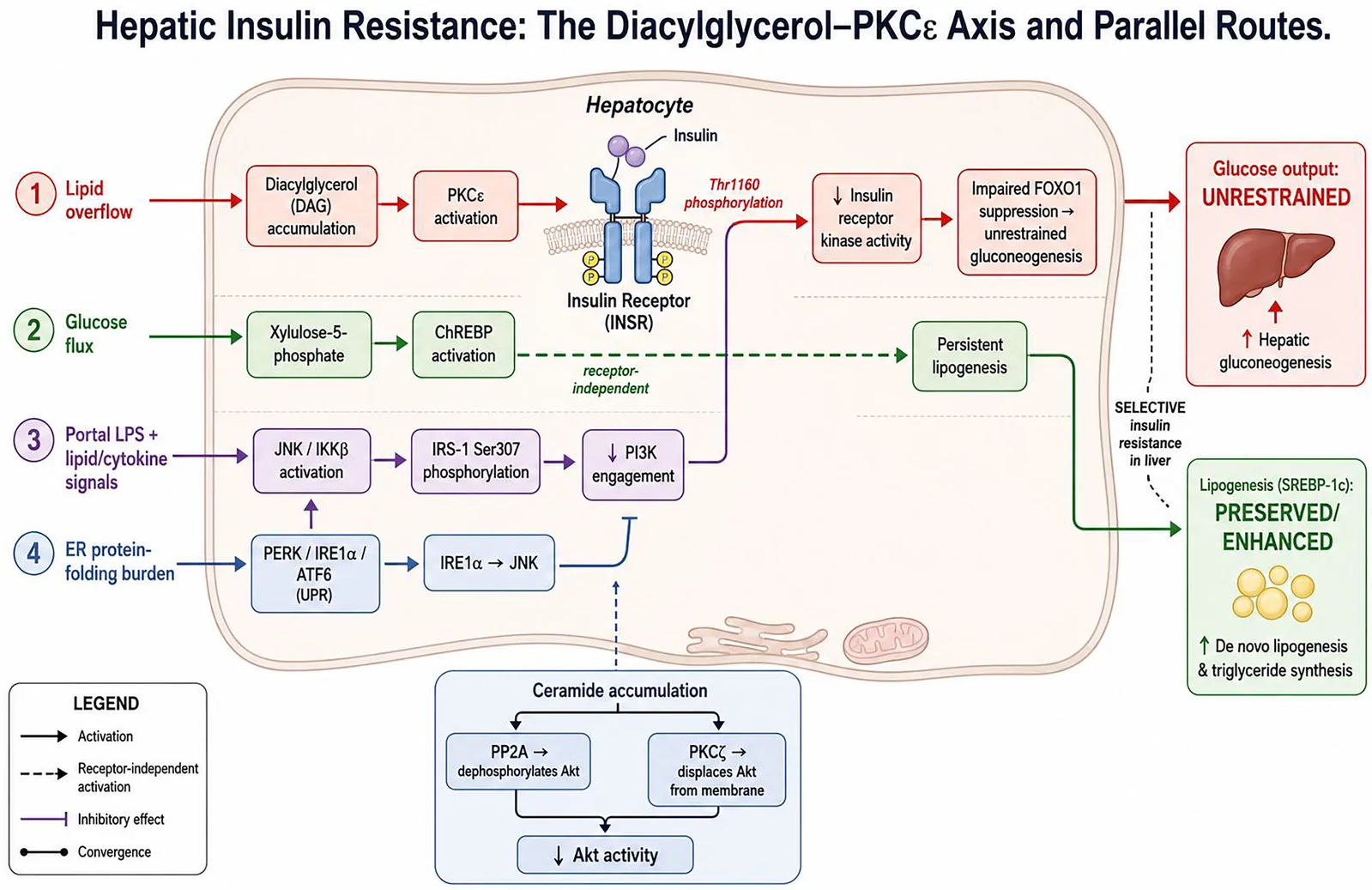
Convergent and parallel signaling routes underlying hepatic insulin resistance. Diacylglycerol-driven PKCϵ activation acts at the insulin receptor itself (Thr1160), explaining impaired gluconeogenic suppression while leaving Akt-independent, ChREBP-driven lipogenesis intact — the basis of “selective” hepatic insulin resistance. In parallel, inflammatory (JNK/IKKβ) and endoplasmic-reticulum-stress (IRE1α) signaling converge on inhibitory IRS-1 serine phosphorylation, while ceramide accumulation acts further downstream via PP2A and PKCζ to blunt Akt directly. Solid mechanistic steps shown here are experimentally supported; the relative weighting of the parallel routes converging on IRS-1 is interpretive and is not established quantitatively.

A second hepatic node operates independently of insulin receptor signaling altogether. Carbohydrate-response element-binding protein (ChREBP) is activated directly by glucose-derived metabolites, particularly xylulose-5-phosphate, and drives glycolytic and lipogenic gene expression in parallel with, rather than downstream of, SREBP-1c (20). Because ChREBP activity tracks substrate availability rather than insulin receptor occupancy, it provides a route by which hyperglycemia itself — the downstream consequence of hepatic and peripheral insulin resistance — feeds back to sustain hepatic lipogenesis even as insulin’s own signal to the receptor is failing, a feed-forward loop that helps explain why hepatic steatosis tends to worsen rather than plateau once glucose control deteriorates.

Chronic hepatic lipid accumulation also engages inflammatory and stress kinases that converge on IRS-1 from a different angle than PKCϵ. Diacylglycerol and ceramide accumulation, together with Kupffer cell activation and lipopolysaccharide exposure via the portal circulation, activate c-Jun N-terminal kinase (JNK) and inhibitor of κB kinase β (IKKβ), both of which phosphorylate IRS-1 on inhibitory serine residues (Ser307 and related sites), reducing IRS-1’s capacity to engage PI3K even when the receptor itself remains intact (21, 22). Ceramides additionally activate protein phosphatase 2A (PP2A), which directly dephosphorylates and inactivates Akt, and promote Akt sequestration away from the plasma membrane through PKCζ-dependent mechanisms, providing a lesion further downstream than either the DAG–PKCϵ or the JNK/IKKβ–IRS-1 routes (23, 24).

The endoplasmic reticulum unfolded protein response contributes a further layer. Chronic nutrient and lipid oversupply increases the protein-folding burden in hepatocytes, activating the PERK, IRE1α, and ATF6 arms of the unfolded protein response. IRE1α in particular activates JNK directly, adding endoplasmic reticulum stress as a third upstream route into the same receptor-distal bottleneck already engaged by lipid- and cytokine-driven signaling (23, 25). **Table 1** summarizes these hepatic signaling lesions, the level of the signaling cascade at which each acts, and the primary experimental or clinical evidence supporting each mechanism. What emerges from considering these routes together is that the liver does not have one insulin-resistance mechanism but several, active at different points in the same cascade and driven by different upstream stimuli — lipid overflow acting through PKCϵ at the receptor, glucose flux acting through ChREBP independently of the receptor, and inflammatory and ER-stress signaling acting through IRS-1 serine phosphorylation and Akt inactivation. Because these routes converge on different points of the IRS–PI3K–Akt trunk, their relative contribution likely shifts as disease progresses, a question taken up directly in Section 10.

**Table 1:** Hepatic insulin resistance: signaling lesions.

Mechanism	Level of signaling cascade	Upstream trigger	Key evidence	Primary source
Diacylglycerol–PKCϵ activation	Insulin receptor (Thr1160)	Hepatic diacylglycerol accumulation from lipid overflow	Human liver biopsy correlation; genetic PKCϵ knockdown normalizes sensitivity independent of steatosis	17–19
ChREBP activation	Transcriptional (glycolytic/lipogenic genes), receptor-independent	Glucose-derived xylulose-5-phosphate	Sustains lipogenesis even as receptor-level insulin signaling fails; specific metabolite trigger contested	20
JNK/IKKβ → IRS-1 Ser307	IRS-1 (receptor-distal)	Lipid- and cytokine-driven inflammation; portal LPS exposure	Classic genetic loss-of-function studies in obese rodent models	21, 22
Ceramide → PP2A/PKCζ	Akt (post-PI3K)	Hepatic ceramide accumulation	Mechanistic studies partly relied on non-physiologic ceramide concentrations; PP2A inhibition paradoxically worsened resistance in one study	23
ER stress (PERK/IRE1α/ATF6)	IRS-1, via IRE1α-JNK	Protein-folding burden from chronic nutrient excess	IRE1α shown to activate JNK directly, linking ER stress to the same distal bottleneck	25

Because this review uses selective hepatic insulin resistance as an organizing device, it is worth stating precisely which parts of it are established and which are not. What is well supported is the phenomenon itself: in resistant liver, suppression of glucose production and stimulation of lipogenesis are dissociated, and this has been documented in both human and rodent studies. What remains unresolved is its mechanistic origin, specifically whether the dissociation is generated within the hepatocyte through branch-selective signaling or is imposed from outside it by substrate delivery, as the lipogenesis findings discussed above suggest. The framework is therefore used here at the level of the observed phenomenon, which is secure, and not at the level of a particular mechanistic explanation for it, which is not. Nothing in the network model developed in later sections depends on which of the two mechanistic accounts proves correct, since both predict the same inter-organ behavior: a liver that exports glucose and triglyceride simultaneously.

It is worth noting that the selective-resistance model itself, though it remains the dominant framework and the one adopted here for organizational purposes, is not as settled as it is often presented. The concept, first articulated clearly by Brown and Goldstein (12), was built substantially on genetic mouse models in which hepatic insulin signaling was manipulated directly. More recent work from the same research group that helped establish the diacylglycerol–PKCϵ mechanism has since reported that isolated, diet-induced hepatic insulin resistance reduces rather than preserves *de novo* lipogenesis in mice, with lipogenesis rebounding only once resistance becomes whole-body rather than liver-specific (13). This suggests that the lipogenic “escape” attributed to insulin signaling may depend substantially on substrate delivery, principally glucose- and fatty-acid-driven flux through ChREBP and direct esterification arriving from the rest of the network, rather than on insulin continuing to signal normally to SREBP-1c within the hepatocyte itself. This does not overturn the broader point that hepatic glucose and lipid handling can be dissociated. It does suggest that where that dissociation is generated — in receptor-proximal signaling within the hepatocyte, or in substrate flux arriving from adipose tissue and the gut — remains unresolved, and it illustrates how a mechanistic model that becomes a field’s default assumption can outrun the evidence supporting it.

## 4 Skeletal muscle: GLUT4 trafficking and the diacylglycerol–PKCθ axis

Skeletal muscle accounts for the majority of insulin-stimulated glucose disposal in the postprandial state, so a defect here has an outsized effect on whole-body glucose tolerance even when the defect itself is modest in molecular terms. Unlike the liver, muscle insulin resistance is not usefully described as “selective”, because muscle’s principal job in response to insulin is a single, well-defined action: translocating GLUT4-containing vesicles to the plasma membrane. Most of the mechanistic literature on muscle insulin resistance is, accordingly, organized around what interrupts that one trafficking step (26, 27).

The core lesion again begins with lipid. Chronic caloric excess and reduced fatty acid oxidative capacity lead to accumulation of intramyocellular diacylglycerol, which activates the novel PKC isoform PKCθ in muscle, paralleling PKCϵ’s role in liver but acting on a different substrate profile. Membrane-associated PKCθ phosphorylates IRS-1 on inhibitory serine residues (Ser1101 among others), reducing IRS-1 tyrosine phosphorylation and, in turn, PI3K activation and downstream Akt signaling to TBC1D4 (26, 27). Because TBC1D4 phosphorylation is the proximate trigger for GLUT4 vesicle exocytosis (Section 2), this single upstream lesion can account for a substantial part of the reduction in insulin-stimulated glucose uptake observed in resistant muscle, though it operates alongside the other lesions described below rather than in isolation. Human infusion studies that acutely raise plasma free fatty acids reproduce this DAG-PKCθ-IRS-1 sequence, and the resulting fall in glucose disposal, within hours (26).

Ceramide accumulation contributes a second, partially independent route. Muscle ceramides activate protein phosphatase 2A, which dephosphorylates Akt directly, and promote translocation of atypical PKCζ to the plasma membrane, where it displaces Akt from its activated membrane pool, a mechanism distinct from the DAG-PKCθ-IRS-1 route because it acts downstream of PI3K rather than at the level of receptor-proximal signaling (23, 24). This distinction has practical consequences for how muscle insulin resistance should be interpreted in any given study: a muscle biopsy showing normal IRS-1 tyrosine phosphorylation but reduced Akt activity points toward the ceramide-PP2A/PKCζ route, while reduced IRS-1 phosphorylation itself points toward the DAG-PKCθ route, and the two are not distinguishable by glucose clamp data alone.

A further complication, and one of the more genuinely counterintuitive findings in this literature, is that total intramyocellular lipid content correlates poorly with insulin resistance across studies, because it is lipid droplet architecture rather than lipid quantity that predicts the metabolic outcome. Endurance-trained athletes carry intramyocellular lipid stores comparable to or exceeding those seen in type 2 diabetes (T2D), yet remain highly insulin sensitive, an observation sometimes called the athlete’s paradox. The resolution appears to be subcellular localization. In trained muscle, lipid droplets are small, numerous, and positioned close to mitochondria in oxidative type I fibers, supporting rapid turnover and oxidation. In insulin-resistant muscle, lipid accumulates in larger, poorly turned-over droplets in type II fibers with reduced mitochondrial density, favoring accumulation of the DAG and ceramide species that activate PKCθ and PKCζ rather than their oxidative disposal (28, 29). This reframes muscle insulin resistance as, at least in part, a defect in lipid flux and mitochondrial oxidative capacity rather than a defect in lipid storage per se, and it explains why interventions that increase muscle oxidative capacity, most notably exercise training, improve insulin sensitivity substantially faster than they reduce total intramyocellular lipid content.

The lipid measurements underlying much of this literature carry an important interpretive limit. Diacylglycerols and ceramides are not single entities but families of species differing in acyl chain length and saturation, and they are distributed unevenly between membrane, cytosolic, and lipid droplet compartments. Total tissue pools therefore aggregate species and compartments with different, and in some cases opposing, signaling activity, which is one reason total measurements correlate inconsistently with insulin sensitivity while compartment- and species-resolved measurements perform better. Studies that resolve DAG stereochemistry, acyl composition, and subcellular localization, or that pair lipid measurement with direct assessment of PKC activation, support the mechanistic conclusions drawn here considerably more strongly than those reporting total pools alone. Where this review refers to DAG or ceramide accumulation, it should be read as shorthand for the species- and compartment-resolved signal rather than for bulk tissue content.

**Table 2** summarizes these muscle-specific lesions alongside their level of action in the signaling cascade. Considered against the liver mechanisms in Section 3, a pattern becomes visible that recurs across every tissue examined in this review: the same two upstream signals, diacylglycerol acting through a tissue-specific PKC isoform and ceramide acting through PP2A and PKCζ, reappear with different downstream consequences depending on which distal Akt substrate matters most for that tissue’s physiological job. The recurrence is systematic rather than accidental — Section 9 returns to it directly when considering the shared molecular machinery, principally post-translational modification and organelle contact-site biology, that makes this cross-tissue repetition possible.

**Table 2:** Skeletal muscle insulin resistance: signaling lesions.

Mechanism	Level of signaling cascade	Upstream trigger	Key evidence	Primary source
Diacylglycerol–PKCθ → IRS-1 Ser1101	IRS-1 (receptor-distal)	Intramyocellular diacylglycerol	Human free-fatty-acid infusion reproduces the sequence within hours	26, 27
Ceramide → PP2A/PKCζ	Akt (post-PI3K)	Muscle ceramide accumulation	Distinguishable from the PKCθ route by normal IRS-1 phosphorylation with reduced Akt activity	23, 24
Lipid droplet architecture and mitochondrial oxidative capacity	Substrate flux/lipid turnover, upstream of DAG and ceramide generation	Reduced oxidative capacity; poor lipid droplet turnover in type II fibers	“Athlete’s paradox”: total intramyocellular lipid content does not predict insulin sensitivity; droplet size, number, and mitochondrial proximity do	28, 29

## 5 Adipose tissue: the lipolysis–adipokine axis and macrophage crosstalk

Adipose tissue occupies a distinctive position in this network: it is a comparatively minor site of insulin-stimulated glucose disposal, but it is the tissue whose failure most directly determines the lipid and inflammatory signals that drive insulin resistance elsewhere. Insulin’s dominant action in adipose tissue is not glucose uptake but suppression of lipolysis, and adipose insulin resistance is, at its core, a failure of that suppression: hormone-sensitive lipase and adipose triglyceride lipase escape insulin’s restraint, free fatty acid efflux rises, and the liver and muscle downstream inherit a lipid load their own signaling networks were not designed to absorb (30, 31). Longitudinal cohort data indicate that this failure of adipose insulin action can be detected before measurable hepatic or muscle insulin resistance appears, consistent with adipose tissue functioning as an early sentinel in the network rather than a late casualty of it (3, 4).

The proximal signaling lesion parallels the ceramide- and DAG-dependent mechanisms already described in liver and muscle, converging on phosphodiesterase 3B and the lipid droplet-associated protein ABHD15, both of which are Akt substrates that normally restrain lipolysis when phosphorylated; ceramide-driven PP2A activation and PKCζ-mediated Akt displacement blunt this restraint in the same way they blunt glucose uptake signaling in muscle (23, 31). What distinguishes adipose tissue mechanistically is less the proximal lesion itself than what happens once lipolysis escapes control: the tissue’s secretory profile shifts in a way that actively propagates insulin resistance outward.

Healthy, insulin-sensitive adipose tissue secretes adiponectin, which acts on AMPK and PPARα in liver and muscle to promote fatty acid oxidation and improve insulin sensitivity in those distal tissues; as adipose tissue becomes dysfunctional, adiponectin secretion falls, removing this protective input at precisely the moment hepatic and muscle tissues are receiving more free fatty acid than they can safely oxidize (32). Simultaneously, expanding and increasingly hypoxic adipose tissue recruits and polarizes macrophages toward a pro-inflammatory phenotype; these macrophages, along with stressed adipocytes themselves, secrete tumor necrosis factor-α and interleukin-6. Both activate JNK and IKKβ locally within adipose tissue and, once circulating in sufficient concentration, in liver and muscle as well, reaching the same receptor-distal serine residues on IRS-1 implicated in Sections 3 and 4 (33, 34). This macrophage-adipocyte crosstalk is bidirectional and self-reinforcing: inflammatory cytokines further impair adipocyte insulin signaling and lipid storage capacity, which increases free fatty acid efflux and further promotes macrophage recruitment, a feed-forward loop that plausibly explains why adipose tissue inflammation, once established, tends to be difficult to reverse with modest weight loss alone.

A further layer of adipose-specific biology involves the tissue’s limited capacity for healthy expansion. When subcutaneous adipose depots reach their expansion capacity, typically through impaired adipocyte hyperplasia and angiogenesis, excess lipid is redirected to visceral depots and to ectopic sites, including liver and muscle, a phenomenon sometimes termed adipose tissue expandability failure. Because visceral adipose tissue is itself more prone to hypoxia, macrophage infiltration, and inflammatory cytokine output than subcutaneous depots, this redirection compounds rather than merely redistributes the metabolic burden, and it links adipose tissue’s own signaling failure directly to the ectopic lipid deposition that drives the hepatic and muscle mechanisms described in Sections 3 and 4 (30). **Table 3** summarizes the principal adipose signaling lesions and their downstream consequences for other tissues in the network. **Figure 3** depicts these three parallel channels together.

**Table 3:** Adipose tissue insulin resistance: signaling lesions and downstream consequences.

Mechanism	Level of signaling cascade	Upstream trigger	Downstream consequence for other tissues	Primary source
PDE3B/ABHD15 lipolysis escape	Akt substrate	Ceramide-driven PP2A/PKCζ activation	Increased free fatty acid efflux to liver and muscle	31
Adiponectin secretion decline	Secretory/endocrine	Progressive adipocyte dysfunction	Loss of AMPK-mediated protective signaling in liver and muscle	32
Macrophage–adipocyte crosstalk	Paracrine inflammatory (TNF-α, IL-6)	Adipose hypoxia and adipocyte stress	Activates JNK/IKKβ locally and, once circulating, in liver and muscle	33, 34
Adipose expandability failure	Tissue-level/structural	Impaired hyperplasia and angiogenesis in subcutaneous depots	Redirects lipid to visceral and ectopic sites, compounding hepatic and muscle lesions	30

**Figure 3:**
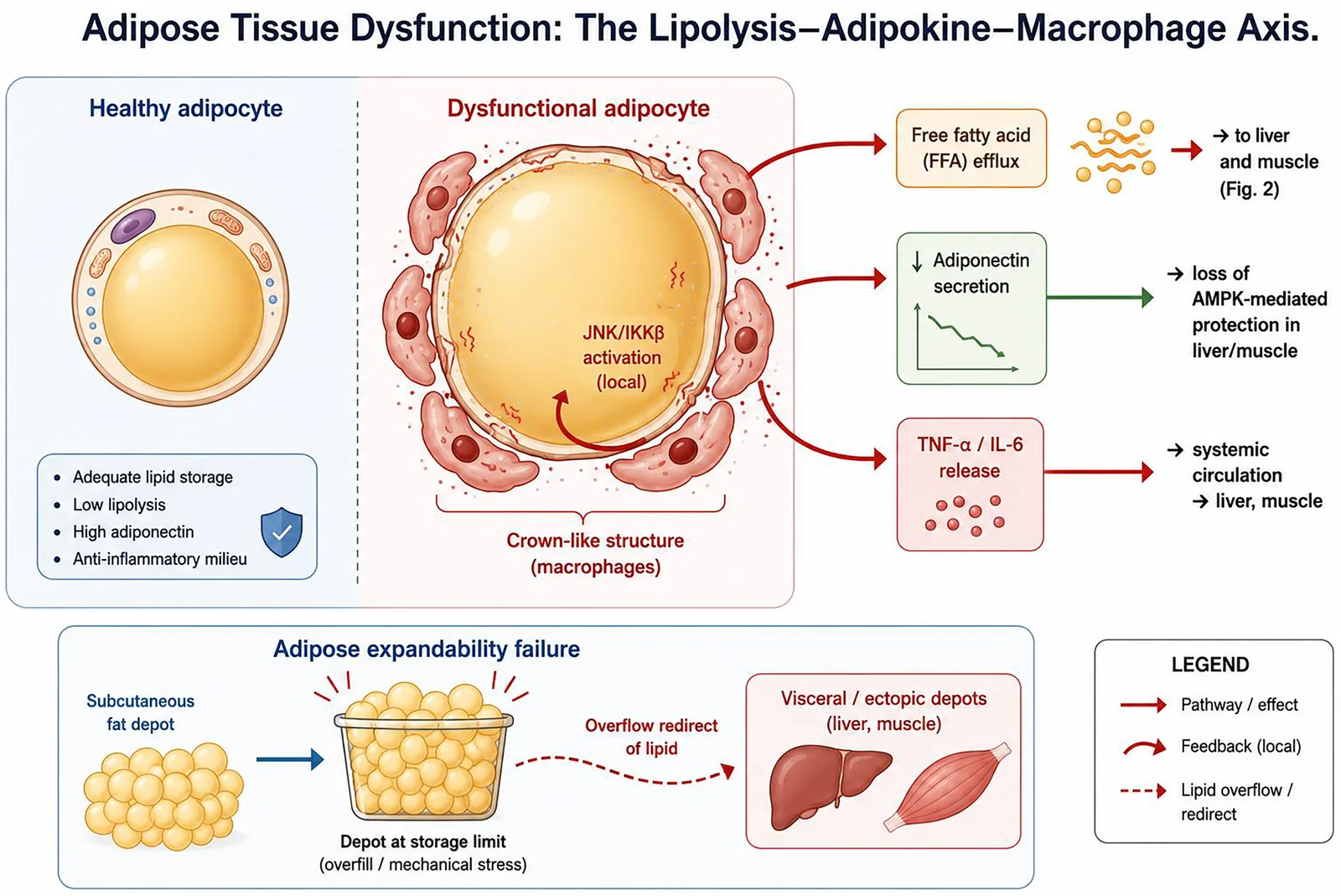
Adipose tissue dysfunction propagates insulin resistance through three parallel channels. Escaped lipolysis (via ceramide-driven PP2A/PKCζ inhibition of PDE3B/ABHD15) increases free fatty acid efflux; declining adiponectin secretion removes a protective AMPK-mediated signal from liver and muscle; and macrophage infiltration, forming crown-like structures around stressed adipocytes, generates TNF-α/IL-6 that engages the same JNK/IKKβ machinery active in liver. When subcutaneous depot expansion fails, lipid is redirected to visceral and ectopic sites, compounding the burden on other tissues. The three channels shown are individually supported experimentally; their relative contribution to systemic insulin resistance in humans is not established.

## 6 Pancreas: β-cell compensation and its limits

The pancreas occupies a different role in this network than the three tissues considered so far. Liver, muscle, and adipose tissue are targets of insulin action whose own signaling failure contributes to systemic resistance; the pancreatic β-cell is, instead, the tissue that responds to that resistance, and whether it responds successfully is what determines whether peripheral insulin resistance remains a silent, compensated state or becomes the hyperglycemia that defines clinical diabetes. This distinction is not merely definitional. It means that any account of insulin resistance organized only around insulin’s target tissues is necessarily incomplete, because it omits the mechanism that determines the clinical consequence of everything described in Sections 3 through 5.

In the earliest phase of peripheral insulin resistance, β-cells compensate through two coordinated mechanisms: increased secretory output per cell, achieved partly through increased sensitivity of the glucose-stimulated insulin secretion (GSIS) machinery to ambient glucose, and increased β-cell mass, achieved through a combination of hypertrophy and, to a lesser extent in adult humans than in rodent models, proliferation (5). Quantitative human autopsy data bear on the limits of this compensation: relative β-cell volume is higher in obese than lean non-diabetic individuals but is reduced by roughly 40-60% in impaired fasting glucose and established T2D, with increased β-cell apoptosis rather than failed replication accounting for the deficit (35). This compensatory hyperinsulinemia is often sufficient to normalize glucose tolerance for years (36, 37), which is precisely why peripheral insulin resistance frequently precedes any clinical glucose abnormality by a substantial margin and why fasting glucose is a late and relatively insensitive marker of the network-level dysfunction this review is concerned with.

Compensation eventually fails, and the mechanisms of that failure mirror, with tissue-specific variations, the lipotoxic, inflammatory, and ER-stress pathways already described in liver, muscle, and adipose tissue. Chronic demand for insulin synthesis places sustained load on the β-cell’s proinsulin-folding machinery, and hyperglycemia and elevated free fatty acids together exacerbate that load, activating the same PERK, IRE1α, and ATF6 arms discussed for hepatocytes in Section 3. In β-cells, however, chronic UPR activation does not merely desensitize insulin signaling; it can trigger CHOP-mediated apoptosis specific to secretory cells under sustained proinsulin misfolding stress (25, 38). In parallel, chronic free fatty acid exposure, particularly to saturated species, impairs GSIS directly and promotes ceramide-dependent β-cell apoptosis through mechanisms analogous to the ceramide-PP2A axis described in muscle. Islet-resident and infiltrating macrophages also secrete interleukin-1β in response to chronic hyperglycemia and lipid excess, creating local islet inflammation that impairs β-cell function independent of systemic inflammatory tone. In isolated human islets, high glucose itself induces β-cell IL-1β production, with downstream NF-κB activation, impaired secretion and apoptosis that are prevented by IL-1 receptor antagonism (38, 39).

A mechanism specific to the β-cell, and one that has reframed how β-cell failure in T2D is understood over the past decade, is dedifferentiation rather than simple apoptotic loss. Under chronic metabolic stress, a substantial fraction of β-cells appear to lose their mature identity, downregulating FOXO1-dependent maintenance of β-cell-specific transcription factors and reverting toward a less differentiated, endocrine-progenitor-like state, or, in some models, transdifferentiating toward an alpha-cell-like phenotype (40, 41). This is functionally distinct from cell death because it is, at least in principle, more readily reversible, which has made it an active target for strategies aimed at restoring rather than merely preserving β-cell function. **Table 4A** summarizes these compensatory and decompensatory mechanisms and, because the transition between them is the clearest example in this review of a genuinely staged, time-dependent process, it is revisited directly in Section 10.

**Table 4A:** Pancreatic β-cell compensation and failure, and cross-tissue convergence machinery.

Stage	Mechanism	Molecular basis	Primary source
Compensation	Increased glucose-stimulated insulin secretion and β-cell mass	Secretory hypertrophy and hyperplasia	5
Early stress	ER stress/unfolded protein response activation	Chronic proinsulin-folding demand under sustained hyperinsulinemia	38
Failure – apoptosis	CHOP-mediated apoptosis; ceramide-driven lipotoxicity	Sustained UPR activation; chronic saturated free fatty acid exposure	38, 5
Failure – dedifferentiation	FOXO1 loss; reversion toward progenitor-like or alpha-cell-like state	Chronic metabolic stress, potentially reversible	40, 41

β-cell trajectory from compensation to failure.

How far this dedifferentiation model extends to human disease remains contested, and the distinction matters for how much weight the mechanism should carry here. The strongest causal evidence is from rodents: lineage-tracing in mouse models established that stressed β-cells lose their mature identity and revert toward progenitor- or alpha-cell-like states rather than simply dying (40). Human evidence is more limited and largely cross-sectional; autopsy and isolated-islet studies have reported an increased proportion of hormone-negative or aberrantly localized endocrine cells in T2D, consistent with loss of β-cell identity (41), but such snapshots cannot directly demonstrate the dynamic reversion, or its reversibility, that the rodent lineage-tracing shows, and they are difficult to separate cleanly from outright β-cell loss. The relative contribution of dedifferentiation versus apoptosis in human T2D, whether it also occurs in obesity without diabetes, and how reversible it is in people rather than in mice therefore all remain open questions (42). Dedifferentiation is treated here as a mechanistically plausible and therapeutically attractive contributor to human β-cell failure whose quantitative weight relative to apoptotic loss is not yet settled.

## 7 Gut: incretin signaling and microbiota-derived metabolites

The gut’s role in this network operates through two largely separable channels: enteroendocrine hormone signaling, which links nutrient ingestion directly to both insulin secretion and central appetite circuits, and microbiota-derived metabolites, which act more diffusely on hepatic, adipose, and immune signaling.

Both channels place the gut upstream of several of the tissues already discussed, which is why its inclusion in this review is organizational rather than incidental: a review that treats the gut only as a site of nutrient absorption misses a substantial fraction of the signals that determine how the other five tissues respond to a given nutrient load. Glucagon-like peptide-1 (GLP-1) and glucose-dependent insulinotropic polypeptide (GIP) are secreted by intestinal L-cells and K-cells respectively in response to nutrient ingestion, and both potentiate glucose-stimulated insulin secretion from pancreatic β-cells in a glucose-dependent manner, meaning their action on the pancreas amplifies rather than replaces the direct glucose signal described in Section 6 (43). This incretin effect, the observation that oral glucose provokes substantially more insulin secretion than an isoglycemic intravenous glucose infusion, is measurably blunted in T2D (44), and the mechanism appears to involve both reduced incretin secretion and reduced β-cell sensitivity to incretin signaling as islet dysfunction progresses, linking gut hormone signaling directly to the β-cell decompensation trajectory discussed in Section 6 (43). GLP-1 additionally acts on hypothalamic and hindbrain circuits to suppress appetite and, through vagal afferent signaling, on hepatic glucose output, providing a second route by which gut-derived signals reach the brain node discussed in Section 8, independent of the pancreatic incretin effect (7).

Bile acids add a third gut-derived signaling channel, distinct from both incretin hormones and microbial fermentation products, and one that links this section back to the liver more directly than either. Synthesized from cholesterol in hepatocytes and released into the intestinal lumen after a meal, bile acids act as signaling molecules once they reach the distal small intestine, activating the nuclear farnesoid X receptor (FXR) in enterocytes and hepatocytes and the G-protein-coupled receptor TGR5 (GPBAR1) on enteroendocrine L-cells and, more broadly, on immune and neuronal cells (45, 46).

Intestinal FXR activation induces fibroblast growth factor 19 in humans (FGF15 in mice), which travels to the liver and suppresses further bile acid synthesis while independently improving hepatic insulin sensitivity and suppressing gluconeogenesis, giving bile acids a direct route into the hepatic signaling network described in Section 3 that operates in parallel to, and largely independent of, the DAG-PKCϵ and inflammatory mechanisms discussed there (45). TGR5 activation on L-cells stimulates GLP-1 secretion through a cyclic-AMP-dependent mechanism distinct from the nutrient-sensing GPR43/GPR41 route described above, so that bile acids and short-chain fatty acids converge on the same incretin output through mechanistically separate receptors; pharmacological and genetic gain- and loss-of-function studies in mice established that TGR5 signaling drives intestinal GLP-1 release and improves glucose tolerance (46, 47). Gut bacteria modify this signal substantially: bacterial bile salt hydrolases deconjugate primary bile acids and downstream microbial enzymes convert them to secondary bile acids with different, generally weaker, FXR and TGR5 agonist activity, meaning that microbiota composition shapes not only the short-chain fatty acid signaling described above but the bile acid signal reaching the liver and L-cells as well (45). The bile acid axis carries real clinical weight rather than serving as a minor mechanistic footnote; bariatric surgery produces glycemic benefits that precede and exceed what weight loss alone would predict, and a substantial share of that durable improvement has been attributed to altered bile acid flow and signaling (48). This is one of the clearer examples in this review of a gut-derived mechanism with demonstrated clinical relevance rather than one resting on correlative or mechanistic data alone.

Gut microbiota-derived metabolites constitute a second, mechanistically distinct signaling channel. Short-chain fatty acids, principally acetate, propionate, and butyrate, are generated by bacterial fermentation of dietary fiber. They act on G-protein-coupled receptors including GPR43 and GPR41 on enteroendocrine cells to stimulate GLP-1 secretion, providing one route by which microbiota composition shapes incretin output; mice lacking FFAR2 (GPR43) or FFAR3 (GPR41) show blunted short-chain fatty acid-triggered GLP-1 secretion and impaired glucose tolerance, giving this route direct loss-of-function support (49, 50). At the opposite end of this axis, disruption of gut barrier integrity under conditions of dysbiosis and high dietary fat intake allows translocation of bacterial lipopolysaccharide into the portal circulation, a phenomenon termed metabolic endotoxemia. Circulating lipopolysaccharide then activates Toll-like receptor 4 on hepatocytes, Kupffer cells, and adipose tissue macrophages, engaging the same JNK and IKKβ signaling described in Sections 3 and 5 through a route originating entirely outside the classical insulin-target tissues (51). This gives the gut microbiome a genuinely bidirectional role in the network: a source of protective signals when barrier function and fermentation capacity are intact, and a source of the same inflammatory input already shown to drive hepatic and adipose insulin resistance when they are not. Of the gut-derived signals discussed here, the bile acid axis carries the strongest causal evidence for a link to systemic insulin sensitivity in humans, through the metabolic effects of bariatric surgery; the short-chain fatty acid and metabolic-endotoxemia mechanisms rest more heavily on associative and animal-model data and are presented here as contributory rather than established drivers of human systemic insulin resistance.

Microbiome evidence requires more caution than the other gut mechanisms discussed here, because diet, adiposity, glycemic status, and medication use all shape microbial composition and are themselves associated with insulin resistance, so cross-sectional compositional differences between insulin-sensitive and insulin-resistant individuals cannot establish direction. Two designs partially break this circularity. Transfer experiments into germ-free rodents show that a metabolic phenotype can travel with the microbiota rather than with the host, and in humans, allogenic infusion of intestinal microbiota from lean donors into men with metabolic syndrome improved peripheral insulin sensitivity within six weeks, alongside an increase in butyrate-producing organisms (52). That trial is small and short, and its effects are not uniformly reproduced, but it is interventional rather than observational and therefore carries weight that compositional surveys do not. On current evidence the microbiota is best described as a modifiable contributor to insulin sensitivity rather than an established primary cause, and this route should not be given the same evidential standing as the bile acid axis discussed above.

## 8 Brain as an integrated node: hypothalamic signaling and the vagal circuit

The brain is treated in most existing literature as either a downstream victim of peripheral metabolic disease, developing its own insulin resistance as a late consequence of obesity and hyperglycemia, or as a separate topic concerned with cognition and neurodegeneration. Both framings are defensible on their own terms, but both understate a mechanistic point central to this review: the hypothalamus is itself an insulin-responsive tissue with the same core IRS-PI3K-Akt signaling architecture described in Section 2, and its output, transmitted through defined autonomic circuits, directly regulates hepatic glucose production and adipose lipolysis. Central insulin action is therefore not merely a consequence of peripheral insulin resistance; it is one of the nodes that determines it, which is why this review treats the brain as a network participant on the same terms as liver, muscle, and adipose tissue rather than as an epiphenomenon of the disease.

Insulin receptors and IRS-2 are concentrated in the hypothalamic arcuate nucleus, where they act on two functionally opposed neuronal populations: proopiomelanocortin (POMC) neurons, whose insulin-stimulated activity suppresses food intake and, through downstream melanocortin signaling, increases hepatic insulin sensitivity, and agouti-related peptide (AgRP) neurons, whose activity insulin normally inhibits (53). Central insulin signaling additionally acts through a hepatic vagal efferent circuit, first characterized in rodent studies, whereby hypothalamic insulin action suppresses hepatic glucose production largely independent of insulin’s direct hepatocyte-level signaling (Section 3) (6, 7). The liver’s overall glucose output therefore reflects the combined and partially separable contributions of local hepatic insulin resistance and central insulin action transmitted through the vagus. Because this vagal contribution operates independently of the liver’s own insulin signaling, no amount of detail about hepatocyte-intrinsic mechanisms can fully explain whole-body glucose output without also accounting for the brain as a node in its own right. **Figure 4** illustrates this closed loop.

**Figure 4:**
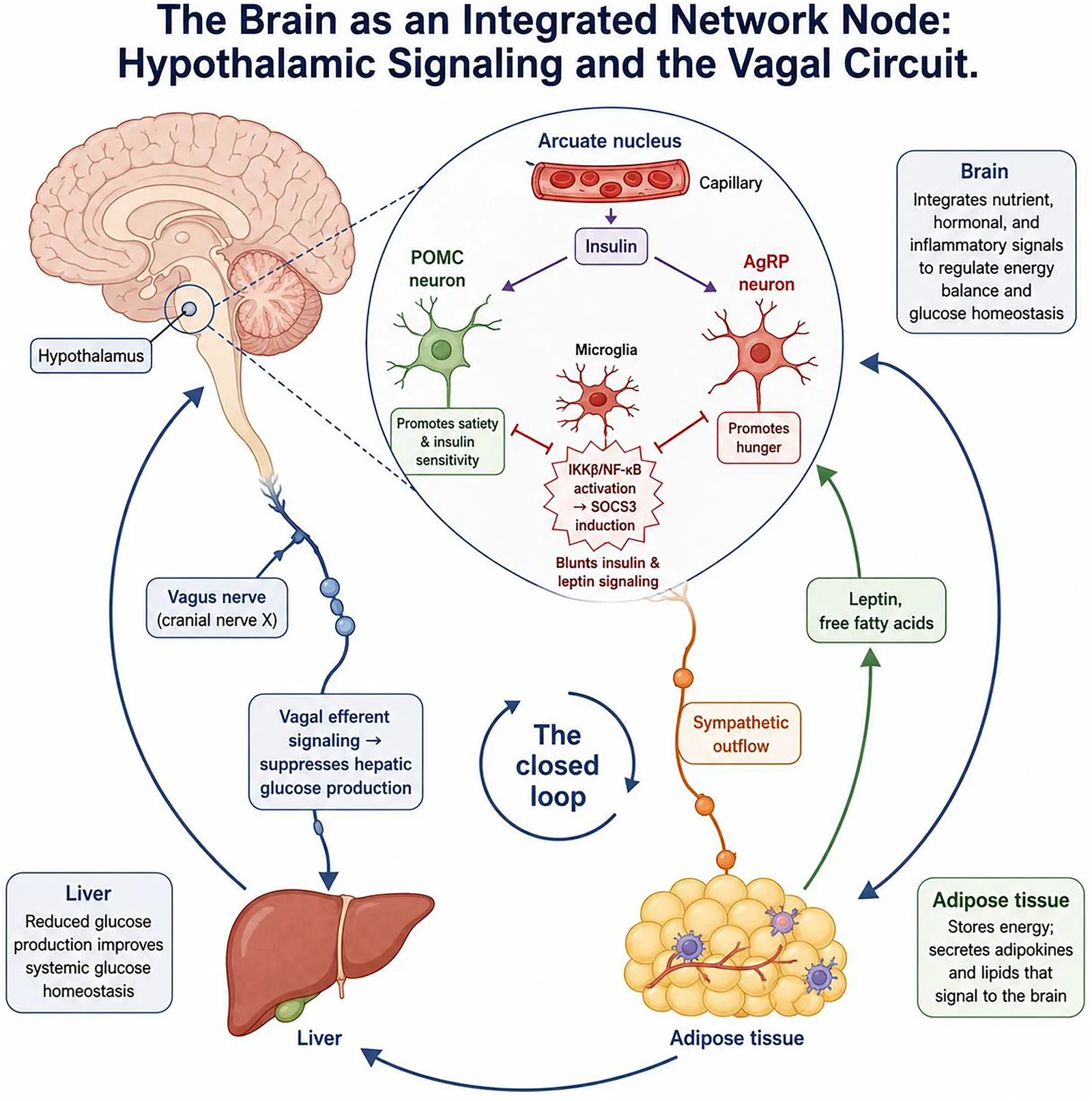
The brain as an integrated node linking adipose dysfunction to hepatic glucose control. Hypothalamic POMC and AgRP neurons receive direct insulin input; their combined output, transmitted via vagal efferents, suppresses hepatic glucose production largely independent of hepatocyte-intrinsic signaling. Hypothalamic microglial activation (IKKβ/NF-κB → SOCS3), triggered by adipose-derived leptin and free fatty acids, blunts this central signaling — closing a self-reinforcing loop between adipose tissue, brain, and liver. Arrows depicting the hypothalamic-vagal-hepatic circuit derive largely from rodent studies; the corresponding human evidence is correlative, and the loop as drawn is a proposed integration rather than an experimentally established circuit in humans.

Central insulin resistance develops through mechanisms that parallel, but are anatomically and molecularly distinct from, the peripheral mechanisms in Sections 3 through 5. Chronic exposure to dietary fat activates microglia in the hypothalamic mediobasal region, driving local IKKβ-NF-κB inflammatory signaling that induces suppressor of cytokine signaling 3 (SOCS3), a feedback inhibitor that binds the insulin receptor and IRS proteins directly and blunts downstream PI3K-Akt signaling in hypothalamic neurons specifically (54). This hypothalamic inflammatory response can be detected within days of high-fat feeding in animal models, substantially preceding measurable peripheral insulin resistance in liver, muscle, or adipose tissue, which has led to the hypothesis that hypothalamic inflammation is not merely a consequence of systemic disease but among its earliest detectable lesions, a possibility with direct implications for the staging framework developed in Section 10 (55). Hypothalamic endoplasmic reticulum stress, engaging the same PERK and IRE1α arms of the unfolded protein response described in Section 3, contributes a parallel route to SOCS3 induction and JNK activation in hypothalamic neurons (54).

Central insulin resistance rarely occurs in isolation from central leptin resistance, and the two share substantial downstream machinery: both insulin and leptin signal through overlapping PI3K pathways in POMC and AgRP neurons, both are antagonized by SOCS3, and hyperleptinemia arising from adipose tissue expansion (Section 5) can itself induce SOCS3 and thereby blunt hypothalamic insulin signaling (54, 55). This provides a direct mechanistic bridge between adipose dysfunction and central insulin resistance, one that requires no change in circulating insulin concentration (54, 55). Considered together with the vagal efferent circuit described above, this closes a genuine loop in the network. Adipose tissue dysfunction (Section 5) elevates circulating leptin and free fatty acids, which impair hypothalamic insulin and leptin signaling. Weakened hypothalamic signaling in turn relaxes vagal suppression of hepatic glucose output, compounding the hepatocyte-intrinsic lesions of Section 3, while reduced central satiety signaling sustains the caloric intake that drove adipose expansion in the first place. Two further qualifications matter for how the hypothalamic node is positioned in this review. First, the evidence that hypothalamic changes appear early does not establish that they initiate the sequence; early appearance is equally consistent with the hypothalamus responding in parallel with other tissues to the same nutrient and lipid excess, and no human study has yet dissociated these possibilities. This review therefore treats hypothalamic involvement as an early and self-reinforcing component of the network rather than as its trigger. Second, it should be emphasized that much of the direct evidence for this loop, particularly its vagal and microglial steps, derives from rodent models; the corresponding human data remain largely correlative, and the quantitative contribution of central insulin resistance to systemic glucose homeostasis in humans is less firmly established than the peripheral mechanisms described in Sections 3 through 5. Section 10 takes up this loop directly when considering how compensation gives way to decompensation over time.

## 9 Convergence machinery: post-translational modification and organelle contact sites

Sections 3 through 8 describe six tissues with six distinct physiological jobs, but a close reading of those sections reveals the same small set of molecular tools reappearing in each: serine phosphorylation of IRS-1, ceramide-dependent activation of PP2A and PKCζ, and, more diffusely, stress responses originating at the interface between the endoplasmic reticulum and mitochondria. The recurrence reflects something structural rather than incidental. It reflects the fact that insulin resistance across tissues is built from a shared post-translational and organellar toolkit, deployed differently depending on which distal signaling branch and physiological output matters in a given tissue. Understanding this shared toolkit explains both why interventions aimed at one tissue often have measurable effects in others, and why a purely tissue-by-tissue account would understate how tightly the six nodes are coupled.

Reversible phosphorylation is the most extensively characterized convergence mechanism. Inhibitory serine phosphorylation of IRS-1, at Ser307, Ser1101, and related residues, is the common downstream output of at least four distinct upstream kinases discussed in this review, JNK and IKKβ in liver and adipose tissue (Sections 3 and 5), PKCθ in muscle (Section 4), and S6K1 acting downstream of chronic mTORC1 activation across insulin-sensitive tissues generally (Section 2). Each of these kinases is itself activated by a different upstream stimulus: inflammatory cytokine signaling, lipid-activated PKC, and nutrient-driven mTORC1 feedback respectively (15). Because four mechanistically distinct upstream signals converge on the same handful of IRS-1 serine residues, measuring IRS-1 serine phosphorylation in a tissue biopsy shows that insulin resistance is present without identifying which upstream route produced it. This limitation is worth stating plainly, because it is routinely elided in studies that treat elevated phospho-IRS-1-Ser307 as evidence for a specific mechanism rather than as a shared downstream readout. Attributing a given phosphorylation event to a particular upstream mechanism therefore requires experimental manipulation of that route, through inhibition, deletion, or selective activation, rather than measurement at the shared endpoint; endpoint measurement can establish that convergence has occurred but not which input drove it.

Ubiquitin-mediated proteasomal degradation provides a second, less reversible layer of convergence. Suppressor of cytokine signaling proteins, principally SOCS1 and SOCS3, bind directly to the insulin receptor and to IRS proteins and recruit an E3 ubiquitin ligase complex that targets IRS-1 and IRS-2 for proteasomal degradation, a mechanism already introduced for hypothalamic neurons in Section 8 but equally active in liver and adipose tissue under sustained inflammatory cytokine exposure (56). Because this route depletes the IRS protein pool itself rather than merely modifying its phosphorylation state, its effects are slower to reverse than phosphorylation-based inhibition once the inflammatory stimulus is removed, which may partly explain why chronic, longstanding insulin resistance responds less readily to short-term intervention than resistance of more recent onset, a distinction taken up further in Section 10.

O-linked β-N-acetylglucosamine (O-GlcNAc) modification adds a nutrient-sensing layer specific to states of chronic hyperglycemia. Elevated glucose flux through the hexosamine biosynthesis pathway increases O-GlcNAcylation of IRS-1 and Akt at sites that overlap with, and interfere with, their normal phosphorylation-based regulation, providing a mechanism by which hyperglycemia itself, once established as a consequence of the tissue-specific lesions described in Sections 3 through 6, feeds back to worsen insulin signaling independent of any new lipid or inflammatory insult (57). This is mechanistically analogous to the ChREBP feed-forward loop described for the liver in Section 3, and its existence in muscle and adipose tissue as well indicates that hyperglycemia-driven signaling deterioration is a network-wide rather than liver-specific phenomenon once glucose control has deteriorated sufficiently.

Mitochondria-associated endoplasmic reticulum membranes (MAMs), physical contact sites between the two organelles that regulate calcium transfer, lipid trafficking, and coordinate the unfolded protein response with mitochondrial function, constitute a candidate organelle-level convergence point, and the one for which the mechanistic evidence in insulin resistance is least mature. MAM integrity is altered, though not always in the same direction, in insulin-resistant liver, muscle, and hypothalamic tissue. Because MAMs sit physically at the interface between the ER stress pathways of Section 3 and the mitochondrial oxidative capacity issues of Section 4, they offer a structural explanation for why ER stress and impaired fatty acid oxidation so consistently co-occur across the tissues examined in this review rather than appearing as independent, coincidentally overlapping mechanisms. Direct manipulation of MAM integrity supports a causal contribution rather than a bystander association: disrupting the mitochondrial MAM protein cyclophilin D impairs insulin signaling in mouse and human primary hepatocytes, ER-mitochondria contacts are reduced in the liver of obese and diet-induced insulin-resistant mice, and restoring contacts improves insulin action (58, 59). **Table 4B** summarizes these four convergence mechanisms, the tissues in which each has been documented, and the distinct upstream signals that funnel into each.

**Table 4b:** Convergence machinery operating across tissue boundaries.

Mechanism	Molecular basis	Tissues documented	Primary source
Inhibitory IRS-1 serine phosphorylation	Common downstream output of JNK, IKKβ, PKC isoforms, and S6K1	Liver, muscle, adipose	15
SOCS-mediated ubiquitination	E3 ligase-mediated proteasomal degradation of IRS-1/IRS-2	Liver, adipose, hypothalamus	56
O-GlcNAcylation	Hexosamine biosynthesis pathway flux modifies IRS-1 and Akt directly	Liver, muscle, adipose	57
Mitochondria–ER contact site (MAM) integrity	Altered calcium and lipid trafficking coordinating UPR and mitochondrial function	Liver, muscle, hypothalamus	59

Two limits of the MAM literature should be stated explicitly. Whether MAM remodeling is a primary driver or a downstream consequence is not settled: MAM alterations are induced by lipid loading, ER stress, and mitochondrial dysfunction, all of which are themselves features of insulin resistance, so their presence in resistant tissue is compatible with either causal position. The experimental manipulations cited above establish that MAM integrity is required for normal insulin signaling, which demonstrates necessity, but necessity in an intact cell does not by itself establish that MAM remodeling initiates the resistant state *in vivo*. Nor are the findings uniform across tissues: reported changes in ER-mitochondria contact differ in direction between liver, skeletal muscle, and hypothalamus, and between studies at different disease stages, so a single description of MAM behavior applicable to the whole network is not currently supportable. MAMs are presented here as a structural convergence point that explains the co-occurrence of ER and mitochondrial abnormalities, not as a unified primary lesion.

## 10 A proposed staging model: from compensation to decompensation

The preceding sections describe six tissue-specific lesions and four convergence mechanisms largely as static facts, but the physiological reality is that they do not appear simultaneously, and the order in which they appear has consequences for how the whole network should be understood and, eventually, treated. This section places the mechanisms from Sections 3 through 9 on an explicit time axis, organized into three stages: silent compensation, early decompensation, and established decompensation. We present this staging explicitly as a proposed, hypothesis-generating model rather than an established temporal sequence. Whole-body longitudinal studies in humans do document a broad progression from compensated insulin resistance through failing insulin secretion to overt hyperglycemia (36, 37); however, the tissue-resolved longitudinal human data that would be needed to confirm the specific order in which individual tissue lesions appear do not yet exist, and the sequence proposed here is assembled largely from cross-sectional human studies and animal-model evidence. The staging is necessarily approximate, individual variation in the sequence and pace of tissue involvement is substantial, and much of the human longitudinal data needed to establish precise timing remains incomplete, a limitation revisited in Section 11. But the broad sequence is better supported by converging lines of evidence than the field’s continued tendency to describe insulin resistance as a single, undifferentiated state would suggest. **Figure 5** places these mechanisms on the time axis this section develops.

**Figure 5:**
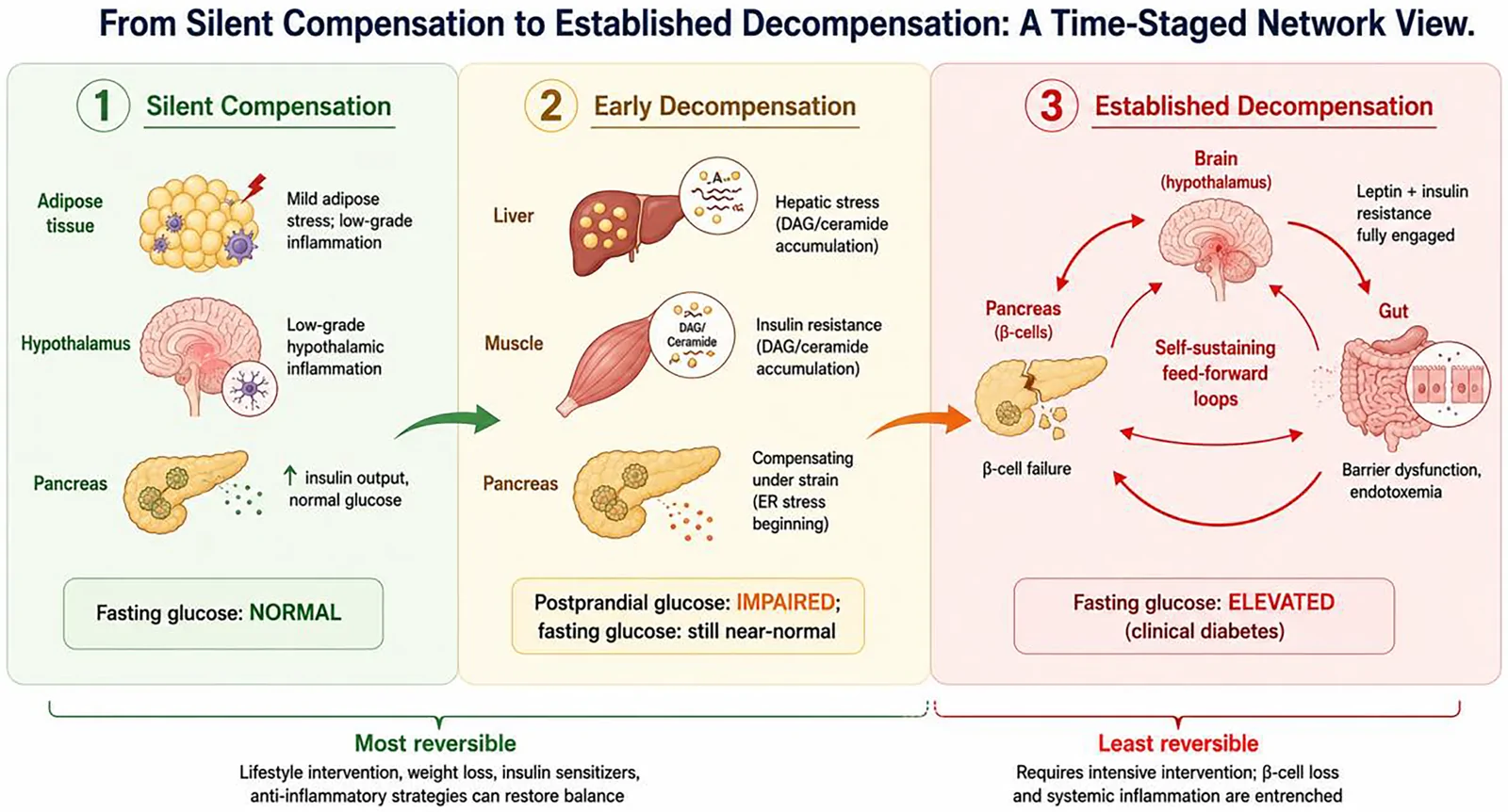
The proposed staged trajectory from silent compensation to established decompensation. Adipose tissue dysfunction and hypothalamic inflammation appear first, while pancreatic β-cells compensate successfully and glycemia remains normal. Hepatic and muscle lesions accumulate next, with postprandial glucose handling affected before fasting glucose. Only once β-cell compensation fails, gut barrier function deteriorates, and central leptin/insulin resistance is fully engaged do the network’s feed-forward loops become self-sustaining — the stage at which clinical diabetes becomes apparent, and at which reversal is hardest to achieve. The staging and the ordering of lesions shown are proposed rather than established; arrows denote hypothesized temporal progression, not demonstrated causal sequence in humans.

Stage one, silent compensation, is characterized by adipose tissue dysfunction as the earliest detectable lesion, consistent with longitudinal cohort data showing that impaired adipose insulin action precedes measurable hepatic and muscle insulin resistance during sustained caloric excess (Section 5). Early hypothalamic inflammatory changes accompany it; in animal models these are detectable within days of dietary challenge, well before peripheral tissues show measurable signaling defects (Section 8). Critically, pancreatic β-cells are, during this stage, doing exactly what they are supposed to do: increasing insulin output to compensate for the earliest peripheral signals, which is precisely why fasting glucose, and even many oral glucose tolerance test results, remain normal throughout this stage despite the network already being measurably perturbed. This is the stage at which the tissue-specific mechanisms described in this review are most amenable to reversal, because the self-reinforcing loops described in Sections 5, 8, and 9, adipose-macrophage crosstalk, the leptin-hypothalamus-vagus-liver loop, and hyperglycemia-driven O-GlcNAc and ChREBP feed-forward signaling, have not yet been substantially engaged.

Stage two, early decompensation, begins as hepatic and muscle signaling lesions accumulate under sustained free fatty acid and inflammatory cytokine exposure from increasingly dysfunctional adipose tissue, engaging the DAG-PKCϵ and DAG-PKCθ mechanisms described in Sections 3 and 4 and the IRS-1 serine phosphorylation convergence point described in Section 9. Compensatory hyperinsulinemia typically remains adequate during this stage, though at a physiological cost: chronically elevated insulin output places sustained proinsulin-folding demand on β-cells, beginning the ER stress trajectory described in Section 6 even while β-cell mass and function remain nominally sufficient to maintain normoglycemia. Glucose tolerance testing may begin to show impairment during oral but not necessarily fasting challenges during this stage, reflecting the fact that postprandial glucose disposal, which depends heavily on the muscle mechanisms described in Section 4, deteriorates before fasting hepatic glucose output, which depends on both hepatocyte-intrinsic signaling and the central vagal circuit described in Section 8, both of which are typically better preserved at this stage.

Stage three, established decompensation, is marked by the transition from β-cell compensation to β-cell failure, whether through UPR-driven apoptosis, ceramide-mediated lipotoxic injury, or dedifferentiation (Section 6), and by the point at which the network’s several feed-forward loops become self-sustaining independent of the original nutritional or inflammatory trigger. Hyperglycemia itself now actively worsens hepatic lipogenesis through ChREBP (Section 3) and worsens signaling broadly through O-GlcNAcylation (Section 9); gut barrier dysfunction and metabolic endotoxemia add a further inflammatory burden that engages the same JNK-IKKβ convergence point already overwhelmed by adipose-derived cytokines, though whether these are a cause or a consequence of this stage remains unresolved (Section 7). Central leptin and insulin resistance are, by this stage, essentially always present, closing the hypothalamic-vagal-hepatic loop of Section 8 in its fully engaged form. It is only at this stage that the clinical syndrome of T2D, as opposed to the compensated insulin resistance that may have been present for a decade or more beforehand, becomes apparent by conventional glycemic testing.

This staging has a direct methodological consequence for how the literature reviewed in Sections 3 through 9 should be read. A substantial fraction of the mechanistic studies underlying this review were conducted in animal models or human cohorts already at stage two or three, when multiple convergence mechanisms are simultaneously active. This makes it difficult to attribute a given phenotype to any single upstream lesion with confidence. A finding that a particular kinase inhibitor improves insulin sensitivity in an established diabetic model, for instance, is not strong evidence that the same kinase is the dominant lesion at stage one, when the loops that amplify its effect at stage three have not yet engaged. This is not a criticism of the individual studies discussed here, most of which are appropriately cautious about the model system in which their findings apply. It is a limitation of the field’s aggregate literature, and it points to precisely the tissue-specific, time-dependent orchestration that remains underexplored despite the mechanistic detail available for each node in isolation.

Two features of this model deserve to be stated as open questions rather than assumptions. The first concerns how the pathways combine. This review describes multiple upstream routes converging on shared endpoints, but convergence does not establish that their contributions add. Partial redundancy is at least as consistent with the evidence: where several routes terminate on the same IRS-1 serine residues, blocking one may be substantially compensated by the others, which would explain why single-kinase inhibition has repeatedly underperformed relative to its mechanistic rationale. Redundancy and synergy are also unlikely to be fixed properties, since the dominant route plausibly shifts with disease stage, diet composition, and tissue. The honest position is that the arithmetic of combination has not been established for any pair of these mechanisms in humans, and the network model presented here does not require it to be additive.

The second concerns variation between individuals. The staging in this section describes a modal trajectory, not an obligatory one. Human cohorts show substantial heterogeneity in which abnormality is detectable first: lean individuals who develop type 2 diabetes tend to present with a secretory defect earlier and with less adipose involvement, individuals with severe obesity more often show adipose and hepatic lesions well before secretory failure, and monogenic and lipodystrophic forms bypass the adipose expansion step altogether while still producing hepatic and muscle insulin resistance. Genetic background, fat distribution, ethnicity, and the composition rather than merely the quantity of nutrient excess all plausibly reorder the early steps. What the longitudinal human data support is the broad endpoint sequence, that compensated insulin resistance precedes secretory failure which precedes hyperglycemia (36, 37); what they do not support is a fixed tissue-by-tissue order applicable to every individual. The staging is therefore best used as a framework for generating testable predictions about typical progression, and as a reminder that mechanistic findings are stage-dependent, rather than as a claim about any particular patient.

## 11 Therapeutic implications by network node

Framing insulin resistance as a network rather than a collection of independent tissue defects has a direct implication for how therapeutic strategies should be evaluated: an intervention’s clinical success is better predicted by how many nodes it engages, and at what stage of the process described in Section 10 it is deployed, than by how selectively it targets any single mechanism described in Sections 3 through 9.

The clearest illustration is the current generation of incretin-based therapies. GLP-1 receptor agonists and the dual GLP-1/GIP agonist tirzepatide were developed as incretin mimetics, extending the pancreatic mechanism described in Section 7, but their clinical effectiveness substantially exceeds what potentiated insulin secretion alone would predict. GLP-1 receptor agonism acts simultaneously on hypothalamic appetite circuits to reduce caloric intake (Section 8); it reduces hepatic steatosis through mechanisms that remain incompletely characterized but appear partly independent of weight loss; and, by reducing adipose tissue mass, it indirectly relieves the lipolysis-adipokine-inflammation axis described in Section 5 (60). Read against the framework developed in this review, these agents are effective largely because they are multi-node interventions that happen to have been discovered through a single-receptor mechanism, not because GLP-1 receptor agonism is uniquely well-matched to any one tissue-specific lesion.

By contrast, therapeutic strategies aimed directly at the convergence machinery described in Section 9, most notably small-molecule JNK inhibitors intended to block the IRS-1 serine phosphorylation shared across liver, muscle, and adipose tissue, illustrate the opposite pattern. Clinical development of direct JNK inhibitors has faced difficulty across multiple disease contexts, and their translation to metabolic disease specifically remains, in the assessment of researchers who have worked on this pathway directly, largely unresolved rather than demonstrably successful (61). The staging framework in Section 10 offers a plausible partial explanation for why a mechanistically well-motivated single-node strategy has been slow to translate: JNK is one of at least four convergent upstream routes to the same inhibitory phosphorylation event, so inhibiting it alone leaves PKC-, S6K1-, and SOCS-mediated routes to the same endpoint intact. Its relative contribution also likely differs across the compensation-to-decompensation trajectory, in ways that trial designs enrolling patients at a single, typically late, disease stage were not positioned to capture (61). This is offered as a hypothesis rather than an established explanation, since publication bias and dosing or selectivity limitations of the specific compounds tested are equally plausible contributors, but it illustrates the kind of question the network framing makes it possible to ask more precisely.

Tissue-targeted strategies further down the network, including thyroid hormone receptor-β agonism aimed specifically at hepatic lipid metabolism, exercise interventions aimed at the muscle mitochondrial and lipid-droplet mechanisms described in Section 4, and approaches to preserve or reverse β-cell dedifferentiation described in Section 6, remain mechanistically well-motivated. With the partial exception of exercise, however, they have generally shown more modest and more variable clinical effects than the multi-node incretin-based approaches. This is consistent with the broader pattern that single-node interventions in a self-reinforcing network face a structural disadvantage once the network has progressed past the earliest stage described in Section 10. **Table 5** summarizes these strategies organized by the network node or nodes each engages, alongside the disease stage at which each is best supported by current evidence.

**Table 5:** Therapeutic strategies organized by network node.

Strategy	Primary network node(s)	Mechanism	Current evidence	Primary source
GLP-1 receptor agonists/tirzepatide	Multi-node: pancreas, brain, adipose, liver	Incretin potentiation, hypothalamic appetite suppression, hepatic steatosis reduction, adipose mass reduction	Weight-independent insulin-sensitizing effect demonstrated	60
Structured exercise training	Muscle	Increases mitochondrial oxidative capacity and favorable lipid droplet turnover	Well-established; improves sensitivity faster than it reduces total intramyocellular lipid	28, 29
β-cell dedifferentiation–reversal strategies	Pancreas	Aim to restore FOXO1-dependent mature β-cell identity	Preclinical; mechanistically motivated by reversibility of dedifferentiation	40
Direct JNK inhibitors	Convergence machinery (single node)	Block IRS-1 serine phosphorylation at its JNK-dependent route only	Clinical translation to metabolic disease remains unresolved; at least three other convergent routes to the same endpoint remain untouched	61

Several agents now in development extend these node-based principles. Among incretin-based approaches, dual and triple agonists push the multi-node logic further: the GLP-1/GIP/glucagon triple agonist retatrutide produced substantially greater weight loss than earlier single- and dual-incretin agents in an early-phase trial, consistent with recruiting an additional glucagon node acting on hepatic lipid handling and energy expenditure (62). Orally available small-molecule GLP-1 receptor agonists are also in late-stage testing as more scalable alternatives to injectable peptides. On the hepatic node, the liver-directed thyroid hormone receptor-β agonist resmetirom improved steatohepatitis and fibrosis in a phase 3 trial and has become the first agent approved specifically for metabolic dysfunction-associated steatohepatitis with fibrosis, offering a tissue-targeted counterpart to the systemic incretin strategies (63). Imeglimin, which has been reported to act on mitochondrial bioenergetics to improve both glucose-stimulated insulin secretion and peripheral insulin action, illustrates a further attempt to engage more than one node with a single molecule; it lowered HbA1c relative to placebo in a phase 3 monotherapy trial (64). These agents sit at varying stages of evidence, but their early trajectory is consistent with the network reading advanced here: the interventions attracting the most clinical interest are those that engage several nodes at once, or that are matched to a specific, well-defined tissue lesion.

The inference drawn here from incretin-based therapies should be stated carefully, because a network reading is not the only explanation available for their performance. Alternative accounts are entirely plausible: these agents produce far larger weight loss than the single-node comparators, and weight loss alone improves insulin sensitivity across every tissue in this review; the trials differ in duration, in the populations enrolled, and in the outcomes prioritized; and the single-node agents that failed were often tested at doses limited by toxicity rather than at doses achieving full target engagement. Head-to-head data help partly, in that they compare agents of the same class under matched conditions. Tirzepatide, engaging both GIP and GLP-1 receptors, produced greater reductions in glycated hemoglobin and body weight than the selective GLP-1 receptor agonist semaglutide in a randomized trial (65), which is consistent with the multi-node reading; but because the additional receptor also produced additional weight loss, that comparison cannot separate node number from weight change either. A related caution applies to how the effects of GLP-1 receptor agonism are apportioned. Potentiation of glucose-dependent insulin secretion and hypothalamic appetite suppression are direct receptor-mediated actions, since the relevant receptors are expressed at both sites. The improvements in hepatic and adipose insulin sensitivity are, by contrast, mostly indirect, following from reduced caloric intake and fat mass, with only a partially weight-independent component reported for hepatic steatosis. This review therefore treats the multi-node interpretation as the most parsimonious reading of the current pattern, not as an established mechanism, and notes that the trials capable of separating node engagement from weight loss, such as weight-matched comparisons of agents engaging different numbers of nodes, have largely not been done.

Because the components of this network rest on markedly different kinds of evidence, **Table 6** grades each one explicitly, separating the strongest available study design from the question of whether the relationship has been established as causal in humans. It is intended to let readers see at a glance which parts of the model are secure and which remain provisional.

**Table 6:** Level of evidence supporting each component of the proposed network.

Network component	Strongest evidence available	Level of evidence	Causal status/caveat
Core insulin signaling cascade (IR-IRS-PI3K-Akt)	Human and rodent tissue, cell systems	Mechanistic; established	Causal (*in vitro*/animal); human confirmatory
Hepatic DAG-PKCϵ axis	Human liver biopsy correlation; rodent knockdown	Human mechanistic + causal animal	Causal in rodents; associative in humans
Selective hepatic insulin resistance (phenomenon)	Human and rodent metabolic phenotyping	Established phenomenon	Observed; mechanism unresolved
Muscle DAG-PKCθ axis	Human lipid infusion studies; rodent models	Human interventional (mechanistic)	Causal in humans for acute lipid exposure
Muscle lipid droplet architecture	Human biopsy, athlete comparisons	Human cross-sectional	Associative
Adipose inflammation (TNF-α, IL-6, macrophages)	Rodent discovery and knockout; human biopsy	Causal animal; human associative	Mixed
β-cell compensation then failure (trajectory)	Longitudinal human cohorts (Pima, Whitehall II)	Human longitudinal	Sequence established at whole-body level
β-cell mass and apoptosis in T2D	Human autopsy quantification	Human cross-sectional	Associative
β-cell dedifferentiation	Rodent lineage tracing; human autopsy	Causal animal; human associative	Contested in humans
Reduced incretin effect	Human physiological studies	Human mechanistic	Established in humans; cause vs consequence unresolved
Bile acid-TGR5/FXR axis	Rodent gain/loss-of-function; human bariatric outcomes	Causal animal; human clinical	Strongest gut route in humans
Short-chain fatty acid-FFAR2/3 axis	Rodent receptor knockouts; human microbiota transfer trial	Causal animal; small human interventional	Contributory
Microbiome composition	Human cross-sectional; germ-free transfer; one human RCT	Mostly associative in humans	Confounded by diet, adiposity, medication
Metabolic endotoxemia (LPS-TLR4)	Rodent infusion; human association	Causal animal; human associative	Direction unresolved
Hypothalamic insulin/leptin action; vagal circuit	Rodent infusion and knockout; limited human imaging	Causal animal; human correlative	Human contribution not quantified
IRS-1 serine phosphorylation as shared endpoint	Cell systems; rodent loss-of-function (S6K1-null)	Causal animal/*in vitro*	Limited direct human causal data
MAM integrity	Mouse and human primary hepatocyte manipulation	Ex vivo/animal causal	Primary vs downstream unresolved
Proposed staging sequence (Section 10)	Whole-body human longitudinal; no tissue-resolved human data	Hypothesis	Proposed; requires validation

## 12 Conclusion

Treating insulin resistance as a network of tissue-specific signaling lesions connected by defined messenger routes, rather than as a single condition with a single mechanism, does more than reorganize an already extensive literature. It clarifies why interventions that engage several nodes at once have outperformed those aimed precisely at a single shared convergence point. It also explains why the earliest and most reversible lesions in this network, in adipose tissue and the hypothalamus, receive comparatively little clinical attention relative to the liver and muscle mechanisms that dominate later in the disease course, and why a mechanistic finding established in one tissue or disease stage cannot be assumed to generalize to another without explicit justification. The tissue-specific mechanisms reviewed in Sections 3 through 8, the convergence machinery reviewed in Section 9, and the staged trajectory from silent compensation to established decompensation reviewed in Section 10 are better understood as facets of a single connected system than as six independent literatures that happen to share a common downstream endpoint.

It is worth stating plainly what this synthesis adds relative to existing reviews of multi-organ insulin resistance, which have generally been organized either tissue-by-tissue or around a single unifying mechanism. Three contributions are intended. First, the review places the tissue-specific lesions on an explicit time axis and states the resulting staging as a falsifiable proposal, with the specific human data that would confirm or refute it identified rather than left implicit. Second, it separates the shared convergence machinery from the tissue-specific lesions, which makes visible a point that tissue-by-tissue accounts obscure: several mechanistically distinct upstream routes terminate on the same small set of molecular endpoints, so a measurement at those endpoints cannot identify the route that produced it, and interventions there face redundancy. Third, it grades the evidence behind each node and edge rather than presenting them as uniformly established, distinguishing human longitudinal, human mechanistic, and animal or *in vitro* support, and marking which relationships remain associative. The framework’s value is that it makes the network’s weak points locatable and its central claim testable, not that it introduces a new mechanism.

The most consequential gap this framing exposes is not a missing molecular mechanism but missing longitudinal, tissue-resolved human data capable of establishing the staging sequence proposed in Section 10 with confidence rather than plausibility. A second gap is the absence of single-cell and spatial approaches able to show whether the convergence mechanisms of Section 9 carry the same relative weight across individual cells within a tissue that they appear to have in bulk measurement. Closing these gaps is unlikely to require new molecular discoveries so much as a shift toward study designs that track the network across tissues and across time in the same patients, rather than characterizing one tissue at one disease stage in isolation, which remains the dominant design in the literature this review has surveyed. **Figure 6** presents this summary map.

**Figure 6:**
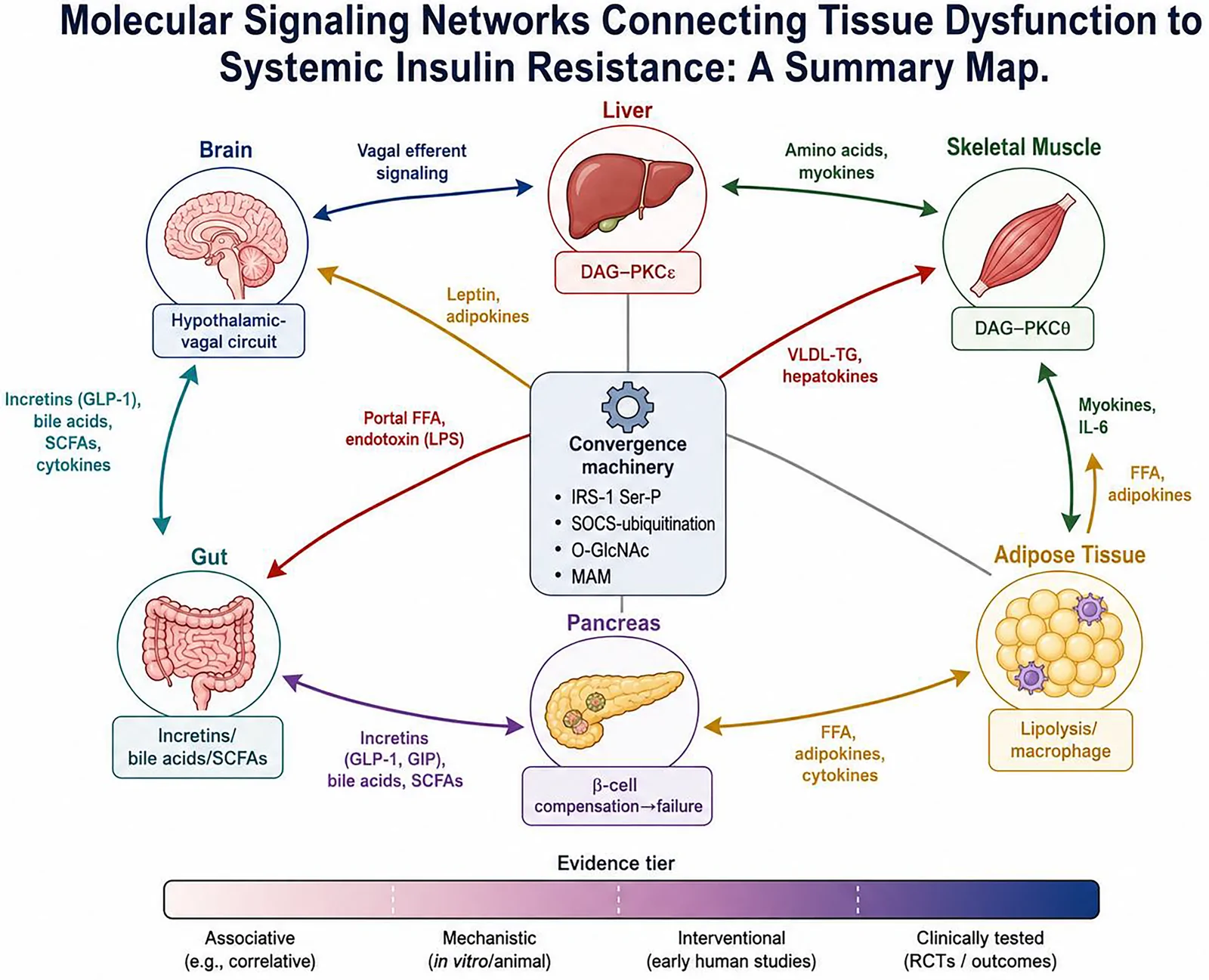
Summary map of the network described in this review. Six tissues, each with a characteristic signaling lesion, are connected by defined messenger routes and share a common convergence machinery (inhibitory IRS-1 serine phosphorylation, ubiquitin-mediated degradation, O-GlcNAcylation, and mitochondria–ER contact sites). Systemic insulin resistance emerges from this connected system rather than from any single tissue or pathway in isolation. Node contents are experimentally supported; the edges summarize proposed inter-organ relationships whose evidential strength varies and is graded in **Table 6**.
